# The Role of APOA-I in Alzheimer’s Disease: Bridging Peripheral Tissues and the Central Nervous System

**DOI:** 10.3390/ph18060790

**Published:** 2025-05-25

**Authors:** Guanfeng Xie, Gege Jiang, Liqin Huang, Shangqi Sun, Yuwei Wan, Fang Li, Bingjie Wu, Ying Zhang, Xiaoyi Li, Bingwan Xiong, Jing Xiong

**Affiliations:** 1 Department of Neurology, Renmin Hospital of Wuhan University, Wuhan 430060, China; 2 Department of Neurology, Union Hospital, Tongji Medical College, Huazhong University of Science and Technology, Wuhan 430022, China; 3 Taikang Center for Life and Medical Sciences, Wuhan University, Wuhan 430000, China

**Keywords:** Alzheimer’s disease, apolipoprotein A1, amyloid beta peptide, lipid metabolism, high-density lipoprotein

## Abstract

Lipid metabolism disorders represent a significant risk factor for the pathogenesis of Alzheimer’s disease (AD). Apolipoprotein E (APOE) has been regarded as a pivotal regulator of lipid homeostasis in the central nervous system (CNS), with polymorphic alleles identified as genetic risk factors for late-onset AD. Despite advances in APOE research and the development of numerous pharmaceutical approaches targeting distinct APOE isoforms, there remain limited treatment approaches for AD that focus on lipid metabolic homeostasis. Consequently, it is necessary to reevaluate the lipid metabolic process in the CNS. Apolipoprotein A1 (APOA-I), a major component of high-density lipoprotein (HDL), plays a crucial role in reverse cholesterol transport from tissues to the liver to maintain lipid homeostasis. Over the past few decades, numerous studies have suggested a connection between reduced APOA-I levels and a higher risk of AD. APOA-I is synthesized exclusively in the liver and intestines, and there is a lack of conclusive evidence supporting its functional significance within the central nervous system, in contrast to APOE, which is produced locally by glial cells and neurons within the CNS. Moreover, APOA-I’s ability to penetrate the blood-brain barrier (BBB) is still poorly understood, which causes its significance in central lipid metabolism and AD pathophysiology to be mainly disregarded. Recent advancements in tracing methodologies have underscored the essential role of APOA-I in regulating lipid metabolism in the CNS. This review aims to elucidate the physiological functions and metabolic pathways of APOA-I, integrating its associations with AD-related pathologies, risk factors, and potential therapeutic targets. Through this discourse, we aim to provide novel insights into the intricate relationship between AD and APOA-I, paving the way for future research in this field.

## 1. Introduction

Alzheimer’s disease (AD) is the most common type of dementia and remains a substantial global economic burden. According to estimates by the World Health Organization, approximately 55 million people worldwide were diagnosed with dementia in 2019, and this number is projected to increase to 139 million by 2050, with a new diagnosis occurring every three seconds [1]. The AD pathology is characterized by two hallmarks: the amyloid plaques consisting of amyloid-β peptide (Aβ) and the neurofibrillary tangles composed of abnormally hyperphosphorylated tau protein [2]. Current treatments for AD provide symptomatic relief by using acetylcholinesterase inhibitors and N-methyl-D-aspartate receptor antagonists, as well as modest disease-modifying effects using disease-modifying treatment (DMT). However, a definitive cure for AD remains elusive [3,4,5]. Recent advancements in the understanding of AD pathology, along with the development of innovative therapeutic strategies, provide promising prospects for the enhancement of treatment efficacy in the future.

**Figure 1:**
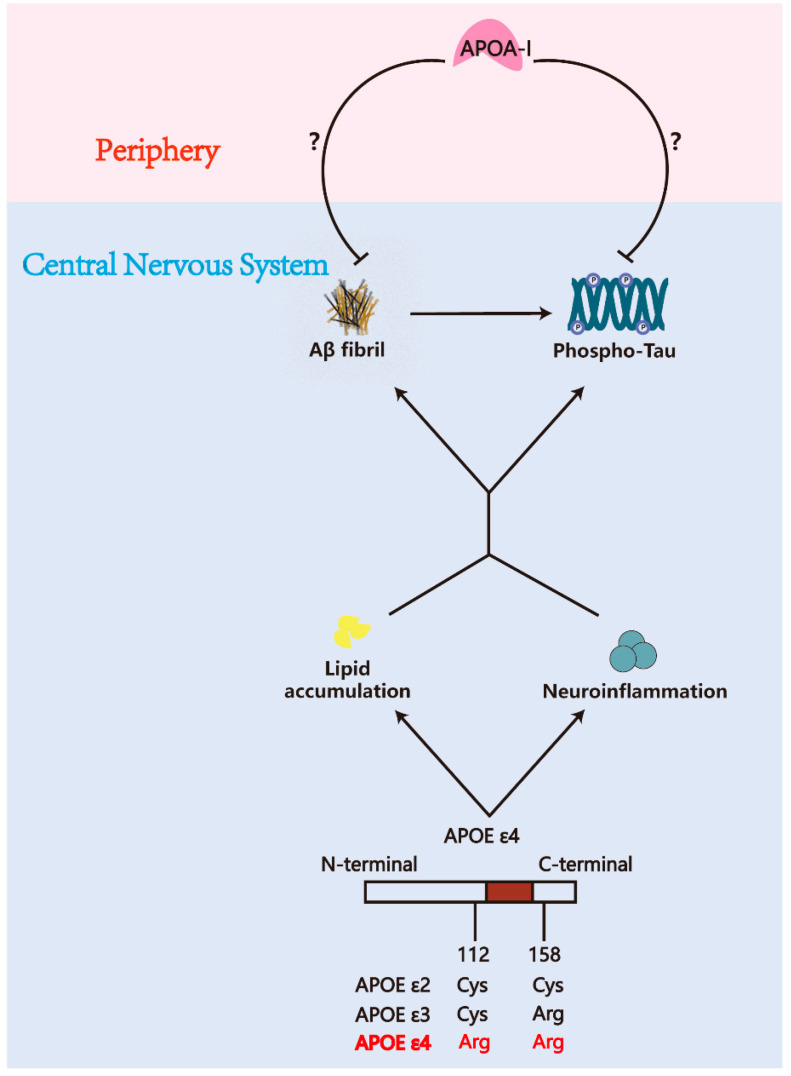
The underlying link between AD pathology and APOA-I. Aβ and tauopathy represent the two core pathological hallmarks of AD. The APOE ε4 allele exacerbates AD progression by disrupting lipid metabolism and amplifying neuroinflammation. While both APOA-I and APOE share conserved functions in peripheral tissues—including high-density lipoprotein (HDL) assembly, reverse cholesterol transport, and anti-inflammatory activity—their cellular origins differ fundamentally within the central nervous system (CNS). Specifically, CNS APOE derives predominantly from glial cells, whereas APOA-I is exclusively synthesized by peripheral organs (liver and intestine). The research has historically concentrated on APOE’s central roles in AD pathogenesis in the past decades. However, emerging evidence now demonstrates that peripherally derived APOA-I can traverse the blood/brain barrier (BBB) and blood/cerebrospinal fluid barrier (BCSFB), which raises interest in the link between AD pathology and APOA-I.

Numerous hypotheses have been proposed to elucidate the pathogenesis of AD, including the amyloid cascade hypothesis, the tau hypothesis, the acetylcholine hypothesis, the neuroinflammation hypothesis, lipid metabolic abnormalities, and the oxidative stress hypothesis, and so on [6,7]. Among these, recent studies have revealed that dysregulated cholesterol metabolism serves as a critical driver of AD progression [8]. In AD patients, elevated cholesterol levels first appear in the early stages of cognitive decline [9]. Additionally, midlife hypercholesterolemia was found to be a substantial risk factor for the progression of AD in a 30-year longitudinal cohort study [10], which has informed subsequent research on the possible use of statin medications for AD prevention and treatment [11]. In addition to upsetting brain homeostasis, the increased cholesterol accelerates the course of AD by inducing neuroinflammation [12]. Therefore, maintaining cholesterol metabolism homeostasis in the central nervous system (CNS) represents a comprehensive therapeutic strategy for AD. Cholesterol clearance in the CNS relies on reverse cholesterol transport (RCT) mediated by high-density lipoprotein (HDL), while low HDL levels have also been confirmed as an established risk factor for AD-related dementia [13,14,15,16,17,18]. These findings collectively suggest that impaired cerebral cholesterol efflux constitutes a pathogenic driver of AD progression, highlighting the maintenance of CNS cholesterol homeostasis as a multifaceted therapeutic strategy against AD. The primary apolipoprotein component of cerebral HDL is apolipoprotein E (APOE) in the CNS, which is synthesized by astrocytes, microglia, and neurons, while the main apolipoprotein in peripheral plasma HDL is apolipoprotein A-I (APOA-I), which is produced by the liver and intestine [13,19]. Given the strong association between the APOE ε4 genotype and an increased risk of AD, and considering that APOE is synthesized within the CNS, a range of pharmacological interventions targeting APOE modification have been developed over recent decades. Few treatments that target APOE have been authorized for clinical investigation despite the fact that numerous preclinical findings have demonstrated that APOE ε4 disrupts the lipid metabolism and immunological stability of the CNS [20,21,22]. APOA-I was previously considered to be an apolipoprotein generated peripherally with limited access to the CNS due to the constraints imposed by the blood/brain barrier (BBB). However, a series of evidence indicates that APOA-I plays a significant role within the CNS. Preclinical research continuously demonstrates the therapeutic potential of APOA-I in reducing AD pathology and improving cognition [23,24,25,26]. Furthermore, reduced levels of APOA-I are strongly associated with AD-related cognitive impairment [27,28]. Recent research has elucidated specific physiological transport mechanisms of APOA-I from peripheral systems into the CNS [29,30]. These findings collectively suggest that the predominant emphasis on APOE may have resulted in a substantial underestimation of the critical role of APOA-I in regulating lipid metabolism within the CNS and its influence on the progression of AD, as shown in Figure 1. Additionally, current perspectives emphasize the necessity of extending mechanistic investigations of neurodegenerative diseases beyond the CNS, as growing evidence underscores the critical contributions of peripheral systems. Approximately 40% of Aβ is transported to the periphery for degradation in the liver and kidneys [31,32,33,34,35,36]. Similarly, nearly 19% of tau protein in the brain flows to the periphery for degradation [37]. Additionally, the pathological protein α-synuclein, which is implicated in multiple neurodegenerative disorders, is also cleared through renal pathways [38,39]. These findings provide substantial theoretical support for the physiological relevance of peripherally derived APOA-I in CNS function and the modulation of AD.

Therefore, elucidating the mechanisms by which APOA-I traverses between peripheral systems and the CNS, along with its modulation of AD pathology, could provide novel insights into AD progression. In this review, we synthesize current knowledge regarding the structure and function of APOA-I while also critically assessing its involvement in AD pathogenesis. Additionally, we summarize the associations between APOA-I and established AD risk factors to evaluate its potential utility in early-stage diagnostics and preventive therapeutics. A more comprehensive understanding of how APOA-I facilitates CNS–periphery crosstalk to influence AD progression may reveal promising avenues for the development of targeted therapeutic strategies.

## 2. APOA-I: Physiological Functions, Transport Pathways, and Association with AD

### 2.1. Metabolism of APOA-I in Peripheral Circulation

**Figure 2:**
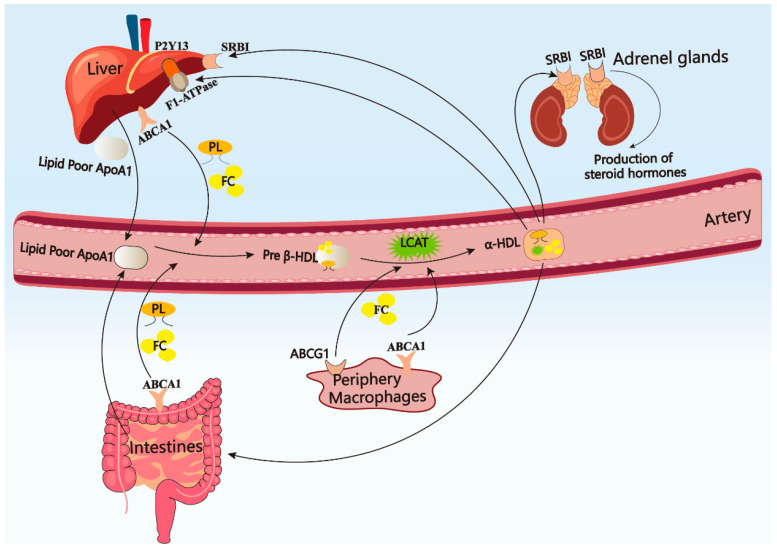
The metabolic process of APOA-I. APOA-I is synthesized and secreted by the liver and intestines, entering the circulation. Then, APOA-I continuously acquires phospholipids (PL) and free cholesterol (FC) from peripheral tissues and macrophages via ABCA1 and ABCG1 receptors, forming pre-β-HDL. Under the action of lecithin/cholesterol acyltransferase (LCAT), the PL from lecithin is transferred to FC, forming cholesteryl esters and promoting the transformation of HDL particles from pre-β-HDL to mature α-HDL, thereby enhancing HDL’s cholesterol transport capacity. Mature HDL is then reabsorbed by the liver via scavenger receptor class B type I (SR-BI), completing the reverse cholesterol transport process [43]. Additionally, HDL can transport cholesterol to the adrenal glands through SR-BI for the synthesis of steroid hormones [45]. HDL also binds to hepatic ecto-F1-ATPase via APOA-I, inducing upregulation of the purinergic P2Y13 receptor to regulate energy metabolism [46].

The pre-form of APOA-I is primarily synthesized in the liver and intestine [40]. The pre-form is then cleaved into mature APOA-I (28 kDa, 243 amino acids) and combines with phospholipids and free cholesterol to form the lipid-free APOA-I via the ATP-binding cassette transporter A1 (ABCA1) [41]. The lipid-free APOA-I is secreted into serum, where it interacts with ABCA1 or ATP-binding cassette transporter G1 (ABCG1) to facilitate the uptake of phospholipids and cholesterol from macrophages in peripheral tissues. This process leads to the formation of discoidal pre-β HDL and spherical α-HDL with the activation of lecithin cholesterol acyl transferase (LCAT) [42]. Ultimately, the mature α-HDL, which contains a substantial cholesterol content, binds to scavenger receptor class B type I (SR-BI) to facilitate the completion of reverse cholesterol transport through hepatic reabsorption or to contribute to steroid hormone synthesis in the adrenal glands [43,44,45]. Apart from SR-BI, HDL can also be endocytosed by the liver through binding to hepatic APOA-I receptors ecto-F1-ATPase [46]. While APOA-I binds to ecto-F1-ATPase, the hepatic hydrolase activity upregulates, and the ecto-F1-ATPase triggers ADP generation via inducing the purinergic P2Y13 receptor upregulation [46]. Additionally, cholesterol derived from APOA-I-formed HDL can be excreted by the intestine through a mechanism known as TICE (Trans Intestinal Cholesterol Efflux) [47]. The metabolic process and physiological function of APOA-I are presented in Figure 2.

The most critical component of HDL is APOA-I, yet the HDL particle core also contains other kinds of apolipoproteins. Most of the apolipoproteins are found in CSF (including APOA-II, APOA-IV, APOJ, APOD, and APOH [48]). Despite the substantially lower concentrations of these apolipoproteins compared to APOA-I, variations in their compositional ratios can significantly modulate HDL functionality [49]. APOA-II is the second most abundant protein component in HDL particles, following APOA-I [50]. However, APOA-II antagonizes the protective effects of APOA-I, leading to diminished cholesterol efflux capacity and reduced anti-inflammatory activity, thereby exacerbating atherosclerosis [51]. Notably, APOA-II redistributed and exacerbated the formation of Aβ fibrils in APOA-I^−/−^ mice [52]. Similar to APOA-II, high levels of APOB generate various proinflammatory compounds that promote the development of atherosclerosis [53]. APOB/APOA-1 is currently recognized as one of the biomarkers for predicting cardiovascular and cerebrovascular disease risk [54,55]. Additionally, recent studies have confirmed that elevated APOB levels serve as one of the risk biomarkers for AD [56,57,58]. These findings suggest that APOA-II and APOB impair the protective effects of APOA-I, and reducing the proportion of APOA-II and APOB in HDL can effectively enhance the protective functions of HDL. Unlike other apolipoproteins, APOD is widely expressed in various tissues, including the CNS, in addition to the intestine and liver [59]. The structure of APOD is distinct from other apolipoproteins, as it lacks the ability to independently assemble into nascent HDL. Its primary function is to facilitate the interaction and binding between HDL and small-molecule lipids [60]. APOD exhibits potent antioxidant capabilities, protecting HDL composed of APOA-I from peroxidative damage [61]. Similar to APOE, the prefrontal cortex exhibits high expression levels of APOD. Moreover, during normal aging, the expression of APOD rises by approximately 5 to 10 times [62]. Interestingly, APOD levels are significantly upregulated in AD, particularly within the hippocampus, entorhinal cortex, pyramidal neurons, and CSF [63,64]. Furthermore, APOD co-localizes with Aβ plaques in the glial system and cerebrovascular units [65]. To date, the mechanistic role of APOD in AD remains incompletely elucidated. Further research in this field may provide novel insights into the impact of HDL on AD pathogenesis. APOJ (also known as clusterin) is also expressed in the CNS and has been shown to co-localize with Aβ fibril in brain tissues, cerebrovascular lesions, CSF, and plasma [66,67,68]. Fernández-de-Retana et al. successfully developed reconstituted HDL containing APOJ (rHDL-APOJ) through the combination of recombinant human APOJ and phospholipids. Their results showed that the developed complex significantly inhibited Aβ fibril formation while enhancing cholesterol efflux capacity and promoting rHDL accumulation in brain tissue [69]. The assembly of HDL exhibits diverse compositional patterns. APOA-I serves as the central structural component, mediating cholesterol reverse transport in peripheral tissues and participating in Aβ clearance within the CNS. However, other core apolipoproteins can differentially modulate HDL functionality. Therefore, research should not focus solely on quantitative changes in HDL levels; a comprehensive analysis of its compositional profile is essential.

### 2.2. The Pathways of APOA-I Transport to the CNS

**Figure 3:**
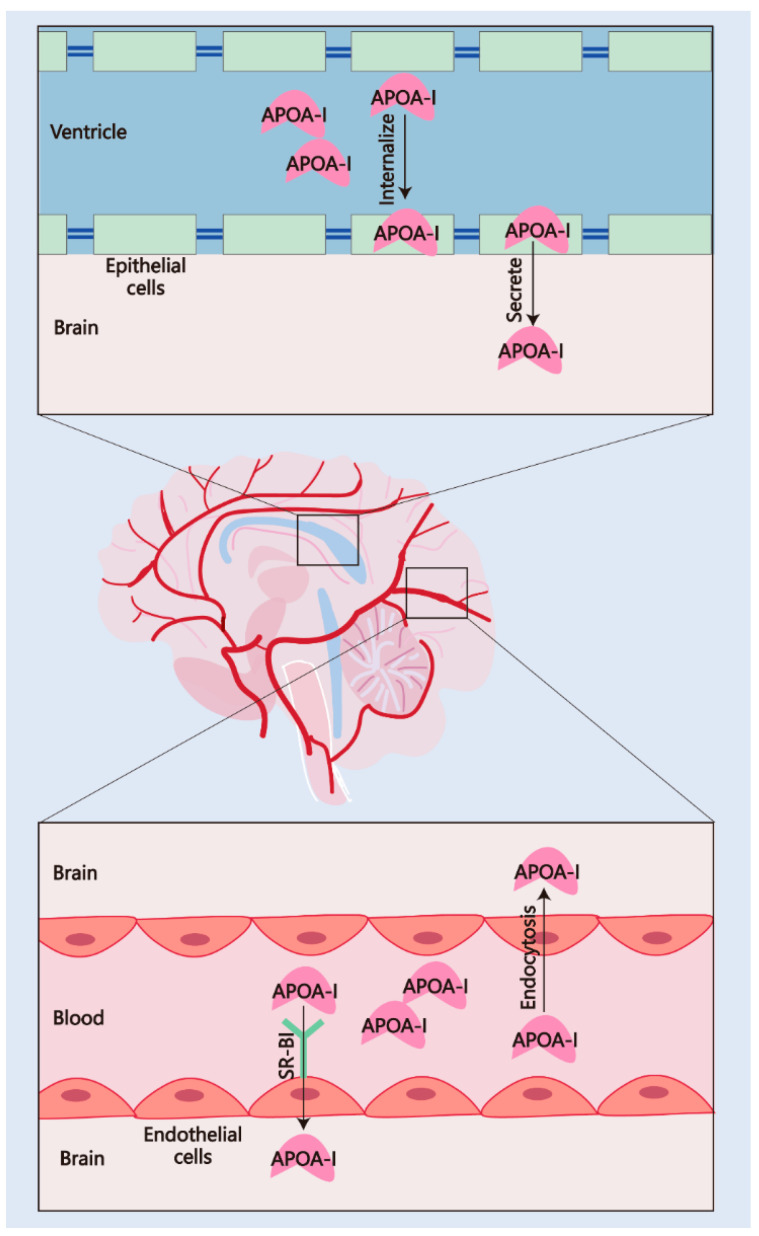
Current perspective of APOA-I transport from the periphery to the central nervous system. APOA-I protein can be detected, but its mRNA is absent within the CNS and the CSF, indicating that APOA-I is entirely synthesized in the periphery and transported into the CNS [29]. The SR-BI on cerebral arterial endothelial cells can act as a receptor mediating the transport of APOA-I across the BBB [73]. Additionally, cerebral microvascular endothelial cells can directly internalize APOA-I through endocytosis [30]. Furthermore, in vivo imaging and tracer techniques have confirmed that APOA-I can be engulfed by choroid plexus epithelial cells and subsequently secreted into the brain parenchyma. However, the specific receptors and mechanisms mediating this BCSFB transport process remain unclear [29].

The CSF levels of APOA-I have demonstrated a strong association with the risk and progression of AD. Two single-center prospective studies have confirmed that low levels of CSF APOA-I are associated with the prevalence of AD and cognitive dysfunction [27,70]. In addition, a decline in CSF APOA-I has been observed at the early stage before the onset of AD symptoms [71]. A meta-analysis study covering 18 studies published between 1992 and 2017 emphasized that low CSF APOA-I levels should be considered a risk factor for AD [72]. These results suggested that APOA-I in the CSF and CNS is significant for AD severity. Given that APOA-I is synthesized entirely in peripheral tissues, understanding the mechanism of its transport from peripheral tissues to the CNS is critical for elucidating AD progression. Thus, a series of studies focusing on APOA-I transportation have been conducted in recent years. The mechanisms of APOA-I crossing the BBB and blood/cerebrospinal fluid barrier (BCSFB) are presented in Figure 3.

#### 2.2.1. Transport via the BBB

The BBB mainly comprises endothelial cells (tight junctions), pericytes, and smooth muscle cells [74]. The BBB demonstrates selective permeability, which is mediated by several receptors. Zhou et al. [30] designed a radioiodinated APOA-I (^125^I-APOA-I) and assessed the permeability and content in different brain regions after intravenous injection in wild-type rats. The ^125^I-APOA-I in the cortex, brain stem, and cerebellum showed higher levels than in the thalamus, which indicates that the BCSFB is not the only channel for APOA-I transportation (the thalamus should reveal greater accumulation when APOA-I is totally transported via the BCSFB due to its periventricular location). Additionally, this research also confirmed that APOA-I can traverse vascular endothelial cells through cholesterol-mediated endocytosis, as evidenced by the transport of Alexa Fluor 647-labeled APOA-I in human cerebral microvascular endothelial cells (hCMECs/D3) [30], elucidating the mechanism of APOA-I transport across the BBB. Another mechanism of APOA-I transport into the BBB is mediated by the HDL receptor SR-BI, which has been proven to exist in brain caveolae capillary endothelial cells and regulates cholesterol movement [73]. Similarly, a recent study reported that SR-BI-mediated transcytosis in brain microvascular endothelial cells is responsible for HDL (mainly formed by APOA-I) transport, and this transcytosis is independent of adaptor protein PDZK1, clathrin, and caveolin-1 (required proteins for HDL uptake in other tissues and cell types) [75]. Remarkably, the interaction of APOA-I-formed HDL and SR-BI was disrupted in AD patients [76]. The expression of SR-BI in astrocytes and vascular smooth muscle within AD brains facilitates the adhesion of microglia and elicits a macrophage response, subsequently promoting Aβ aggregation and contributing to cerebral amyloid angiopathy [77]. These findings reveal the mechanism underlying the close link between CSF APOA-I levels and AD pathology.

#### 2.2.2. Transport via the BCSFB

The BCSFB is formed by the choroid plexus (CP) and regulates the exchange of substances between the CNS and the periphery through CSF [78]. Stukas et al. [29] observed APOA-I accumulated in the CP 30 min after intravenous injection in a saturable, dose-dependent manner. This Alexa Fluor 647-labeled human recombinant APOA-I showed the ability to internalize and transport across confluent monolayers of primary human CP epithelial cells. Interestingly, the 647 fluorescence signal was not detected in the cerebrovascular structures in vivo; however, this human recombinant APOA-I can bind, internalize, and transport across vascular endothelial cells in vitro [29], suggesting that the mechanisms by which APOA-I crosses the BBB and BCSFB are distinct. Despite the surface area of the BCSFB for transporting substances being about 5000 times smaller than the BBB, the significance of APOA-I transport across the BCSFB remains remarkable. Given that the low-density lipoprotein receptor-related protein 1 (LRP-1)-mediated clearance of Aβ across the BBB is impaired in AD [79], the brain may rely more significantly on the reabsorption of interstitial fluid (ISF) Aβ via CSF for its transport across the BCSFB [80]. In AD patients, the CP undergoes structural and functional damage. In detail, the atrophy of epithelial cells leads to a reduction of at least 50% in CSF production. This decline, combined with an increase in ventricular volume, results in reduced CSF flow and abnormal substance exchange [80]. Simultaneously, the clearance of potentially toxic peptides from ISF into CSF is significantly diminished [80,81]. Multiple in vitro studies have confirmed that APOA-I possesses the ability to bind and clear Aβ. Its normal transport across the BCSFB plays a significant role in Aβ pathology. In summary, although transport across the BCSFB may not be the primary route for APOA-I to enter the CNS from the periphery, it plays a significant role in the progression of AD, particularly in modulating Aβ burden.

### 2.3. Clinical Evidence Links APOA-I to AD Pathogenesis

As the primary protein component of plasma HDL, constituting roughly 70% of its protein content, APOA-I has been extensively studied for its functions in modulating lipid homeostasis and its therapeutic potential in cardiovascular disease management [82]. Due to the functional similarities between APOA-I and APOE, the clinical relevance of APOA-I in AD was first investigated decades ago. Early cross-sectional studies revealed significantly reduced serum levels of APOA-I in AD patients [83], and this is independent of APOE genotype [84]. Subsequent studies on biological biomarkers in aging individuals with AD or cognitive decline have further confirmed that low APOA-I levels are strongly associated with cognitive deterioration [85,86,87]. In addition to being significantly lower compared with age- and gender-matched healthy controls, APOA-I levels also demonstrate distinct variations among the AD subjects, with lower concentrations directly correlating with the severity of cognitive impairment (as evidenced by a strong association between APOA-I levels and Mini-Mental State Examination) [88]. Consistent with the reduced peripheral APOA-I levels, CSF APOA-I concentrations are also significantly decreased in AD patients [27,70,71], indicating that the downregulation of APOA-I is closely associated with the onset and progression of AD. On the contrary, Steven D. Harr et al. [89] and Song et al. [90] found that the levels of APOA-I remained unchanged in the brain tissue and CSF of AD patients compared with non-AD subjects. These discrepancies may stem from stage-dependent functional alterations in APOA-I during AD progression. We noticed that although immunoblot analysis showed no difference in APOA-I levels in the frontal lobe between AD patients and control subjects, immunohistochemical staining revealed APOA-I-positive immunoreactivity in occasional senile plaques within the AD cortex, similar to APOE [89], suggesting that although the detected APOA-I levels were not reduced, the APOA-I bound to senile plaques lost its normal physiological function and became incorporated into the AD pathology. While APOA-I can bind Aβ and mediate its immune clearance through the SR-BI receptor [91], overactivated macrophages and microglia may simultaneously trigger neuroinflammation, ultimately contributing to the formation of neuritic plaques [92]. Thus, in this study, postmortem brain tissue analysis revealed that despite maintained APOA-I quantity, its functional integrity was compromised, and it had become pathologically integrated. Additionally, the binding capacity of HDL and APOA-I to both ABCA1 and SR-BI transporters is significantly compromised in AD patients [76], indicating that APOA-I not only exhibits altered expression levels but also demonstrates impaired cholesterol transport capacity in AD patients. In summary, APOA-I demonstrates a significant correlation with cognitive function in AD patients. Current clinical practice relies primarily on subjective neuropsychological assessments for evaluating cognitive impairment, while objective biomarkers remain scarce. Emerging evidence indicates that tau burden rather than Aβ load correlates with cognitive decline in AD. Given the established relationship between APOA-I and cognitive performance, we propose that APOA-I could serve as a complementary biomarker to existing indicators. When integrated with other pathological markers (e.g., p-tau181, p-tau217, and GFAP), APOA-I may enhance the sensitivity and specificity of diagnostic frameworks for both disease staging and cognitive monitoring in AD, which holds particular significance for AD MDT decisions.

### 2.4. APOA-I Polymorphisms and AD

The APOA-I gene is situated on chromosome 11q23-q24 and encodes a single polypeptide chain comprising 243 amino acid residues. To date, public databases have cataloged 48 polymorphisms within the APOA-I gene [93]. Investigations into a common A/G polymorphism at the −75 position within the G/C-rich promoter region of APOA-I have demonstrated that the A allele is associated with a twofold to sevenfold increase in transcriptional activity [94,95,96]. The −75 G to A polymorphism has been confirmed to be associated with severe coronary artery disease and elevated serum levels of low-density lipoprotein following an increased consumption of monounsaturated fats [97,98]. Notably, Vollbach et al. [99] examined the influence of these polymorphisms on AD risk in a cohort comprising 427 AD patients and 500 healthy controls of German and English ancestry. The research found that the A allele of the APOA-I −75 bp G/A polymorphism was linked to a heightened risk of AD in early-onset nonfamilial AD. AD patients who were homozygous for the A allele at the −75 bp position exhibited an onset of the disease eight years earlier than those who had at least one G allele. Conversely, another study that contained three independent European population samples conducted by Helbecque et al. [93] found no impact of the G to A polymorphism at position −75 bp in the APOA-I gene on AD risk. Similarly, a common single-nucleotide polymorphism (SNP) test for the Japanese population and another 3-year prospective study that contained 173 AD patients and 150 healthy controls showed no associations between the APOA-I polymorphisms and the risk of AD [100,101]. What requires our attention is that despite no association being found with AD risks, the G to A polymorphism at position −75 bp increased the risk of cognitive dysfunction [93]. Collectively, the effect of polymorphism at position −75 bp in the APOA-I gene on AD risk remains controversial. Further multicenter, large-scale population data studies are needed. In particular, attention should be focused on the effects of the gene polymorphism of APOA-I on HDL structure and function, lipid metabolism, AD stage and pathology, and cognitive function.

## 3. APOA-I Modifies the Pathology of AD

**Figure 4:**
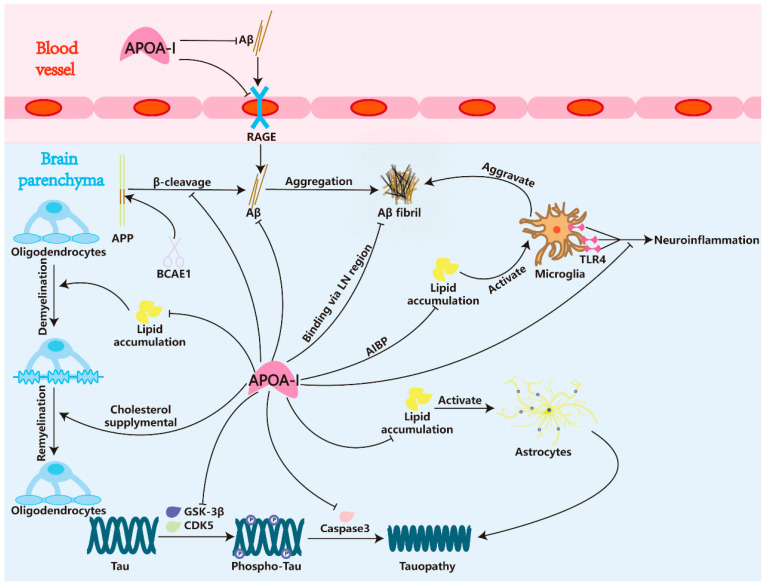
APOA-I is closely related to AD pathology. The level of APOA-I is negatively correlated with the activity of BACE1 [102]. Additionally, L-4F, an APOA-I mimetic, can suppress the expression of the receptor for advanced glycation end products (RAGE), which mediates the transport of peripheral Aβ into the CNS [103]. Furthermore, APOA-I can directly bind to Aβ, reducing its neurotoxicity [104,105,106]. Through its conserved structural sequence LN, APOA-I also binds to Aβ fibrils, delaying further aggregation of tangles [107,108,109]. APOA-I alleviates lipid overaccumulation, thereby reducing microglia- [110,111,112,113,114] and astrocyte-related [112,114] inflammation and oligodendrocyte cell death [115,116,117] while providing lipid substrates for oligodendrocyte remyelination to promote myelin regeneration [118,119]. APOA-I also inhibits tau-related phosphorylation kinases, such as GSK-3β and CDK5, and suppresses caspase-3, an enzyme responsible for tau truncation. However, these changes in tau-related enzymes were observed in non-AD models, and their mechanisms in AD models require further investigation [120,121].

The Aβ and tau hypotheses remain the predominant frameworks for understanding AD pathogenesis. Accumulating evidence demonstrates that APOA-I interacts with both Aβ and tau pathologies. Furthermore, the glial system dynamically modulates lipid metabolic homeostasis, forming bidirectional interactions with Aβ/tau cascades. Within the glial system, APOA-I exerts multifaceted neuroprotective effects through lipid regulation. This section comprehensively reviews the modifying roles of APOA-I in AD pathophysiology. The crosslink between APOA-I and AD pathology is presented in Figure 4.

### 3.1. APOA-I and Aβ

The presence of Aβ is the hallmark of AD, which is characterized by the accumulation of Aβ and the formation of Aβ plaques, also known as senile plaques. These pathological features are accompanied by neurofibrillary tangles (NFTs), synaptic dysfunction, and neurodegeneration. Aβ is produced through a series of proteolytic cleavages of the amyloid precursor protein (APP). The β-secretase (BACE1) cleaves APP at M596, which produces a soluble N-terminal fragment and a C-terminal fragment that remains anchored in the cell membrane. The C-terminal fragment is then cleaved by γ-secretase, which releases the Aβ peptide. The exact length of the Aβ peptide can vary, and the most common forms are Aβ40 and Aβ42. Aβ42 exhibits a higher propensity for aggregation and is implicated in the formation of amyloid plaques [122]. The present known clearance of Aβ in the brain involves the following: (1) Efflux transport: Aβ is able to cross the BBB through receptors in endothelial cells, including the receptor for advanced glycation end products (RAGE) and lipoprotein receptor-related protein 1 (LRP-1). RAGE regulates the influx of circulating Aβ into the brain, while LRP-1 facilitates the efflux of brain-derived Aβ into the circulation [123,124]. (2) Immune phagocyte: Microglia and astrocytes are capable of phagocytosis and clear Aβ [125,126]. (3) Enzymolysis: clearing through brain-degrading enzymes such as neprilysin [127]. (4) Lymphatic efflux: Recent studies have shown that the Aβ efflux from the CNS is mediated by the brain’s lymphatic system. The cerebral lymphatic system facilitates the transport of Aβ in CSF to the cervical lymph nodes for clearance via meningeal lymphatic vessels [81,128,129]. Preventing Aβ accumulation in the CNS is currently the mainstream therapeutic strategy for AD.

APOA-I has been extensively studied in relation to a reduced Aβ burden in AD patients [27,28,71,130,131,132,133]. Given the related association between APOA-I and AD patients, several scientific preclinical studies have been performed to explore the links between APOA-I and Aβ. Interestingly, APOA-I was found to bind to Aβ and reduce the Aβ-induced neurotoxicity in these preclinical studies [104,105,106]. Rebecca Frankel et al. [107] captured the interference of APOA-I on the Aβ42 fibril but not monomer to retard the fibril aggregation through the techniques of surface plasmon resonance, microfluidic diffusional sizing analysis, and cryogenic electron microscopy. Recent investigations have identified that the Aβ-binding region of APOA-I corresponds to the highly conserved N-terminal sequence, 42LNLKLLD48 (‘LN’) [108,109]. APOA-I binds to Aβ through the LN region to form non-covalent complexes, and the complexes were detected within the CSF of AD patients. The interaction with APOA-I affected the aggregation of Aβ and protected hippocampal neurons from Aβ toxicity [104]. In addition to direct binding, the cellular cholesterol metabolism mediated by APOA-I is associated with BACE1. Elham Fanaee-Danesh et al. [102] used retinoid X receptor agonist bexarotene (Bex) and the peroxisome proliferator-activated receptor α agonist astaxanthin (Asx) to disrupt the APP cleavage process in primary porcine brain capillary endothelial cells (pBCECs) and in 3xTg AD mice. Asx/Bex suppressed the transcription/activity of BACE1 and reduced Aβ oligomers, accompanied by an APOA-I increase. However, there is no direct evidence to elucidate the influence of APOA-I on BACE1. APOA-I also exerts influence the Aβ transportation. Specifically, APOA-I mimetic peptide L-4F significantly suppressed the expression of RAGE in the ischemic brain in a type 2 diabetes mellitus (T2DM) mouse model [103]. Furthermore, the human recombinant APOA-I Milano variant (which contains the R173C mutation and confers protection against atherosclerosis development) also decreased RAGE levels in cortical vessels and modified insoluble brain Aβ burden in APP23 transgenic mice [134]. In conclusion, APOA-I ameliorates Aβ toxicity in the brain by preventing the influx mediated by RAGE and directly binding to Aβ fibril to interfere with the aggregation in a conserved region named LN. Additionally, APOA-I showed associations with BACE1, but further confirmation is required.

### 3.2. APOA-I and Tauopathy

Tau is a microtubule-associated protein that maintains the stability of microtubules in neurons and promotes axonal growth. Under physiological conditions, low-phosphorylated tau is localized in the axons of neurons. Neurofibrillary tangles (NFTs), composed of abnormal folding and aggregated tau proteins, are the hallmarks of AD [135]. The burden of tauopathy is closely associated with the severity of clinical symptoms in AD patients rather than Aβ pathology [136]. Targeted tau therapies have shown more significant effects on symptom management in AD [136]. Recently, some studies reported that the CSF APOA-I was negatively correlated with CSF phosphorylated tau (p-tau) levels [70,137]. Nevertheless, the current understanding of the relationship between APOA-I and tauopathy remains limited. In this context, we summarize and discuss the potential mechanisms by which APOA-I may regulate tau pathology.

Guochen Han et al. [138] developed a nanodrug composed of methylene blue, endogenous APOA-I, and its mimicking peptide 4F-fused angiopep-2 (APLN/MB), which effectively reduced p-tau levels by 25.31%. However, HDL containing APOA-I are usually used as a nanocomposite to penetrate the BBB rather than as an active ingredient [139]. Additionally, the APLN/MB promoted Aβ clearance while reducing p-tau [138]. It is difficult to evaluate the therapeutic effect of APOA-I on tauopathy since Aβ is able to drive tauopathy [140,141].

Over the past few decades, significant progress has been made in understanding the role of tau toxicity in the pathogenesis of AD. Studies have shown that post-translational modifications (PTMs) of tau, such as phosphorylation, acetylation, ubiquitination, SUMOylation, and truncation, are involved in its stability, misfolding, aggregation, and degradation. The accumulation of tau protein in cells leads to cognitive deficits, including mitochondrial dysfunction, impaired synaptic plasticity, gliosis, and neuroinflammation through multiple pathways [142]. In AD and the other tauopathies, phosphorylation is one of the most extensively studied PTMs of tau protein. Under physiological conditions, phosphorylation regulates tau’s affinity for microtubules, consequently influencing their stability and assembly. The phosphorylation level of tau is extremely elevated in the aged brain and AD patients [143,144]. Numerous protein kinases are involved in the regulation of tau phosphorylation, with glycogen synthase kinase-3β (GSK-3β) and cyclin-dependent kinase 5 (CDK5) attracting considerable scholarly attention. The co-localization of GSK-3β, CDK5, and pathological tau within the brains of individuals with AD has been widely reported. Both GSK-3β and CDK5 have the capacity to phosphorylate tau at multiple sites associated with AD, including Ser202, Thr212, Ser214, Ser404, Ser217, Thr205, Thr231, Ser235, and Ser396 [145,146,147]. APOA-I showed a negative correlation with GSK-3β and CDK5 signaling in several diseases but not in AD [120,121,148]. Similarly, APOA-I also exhibited a negative correlation with hippocampal caspase 3 (an enzyme that cleaves tau into truncation to promote aggregation, mislocalization, and propagation [149]) in related phenotypes of the autism spectrum disorder model [120]. Regretfully, the correlation between APOA-I and enzymes/kinases mediating PTMs on tauopathy remains limited. Future studies in this field hold significant potential for advancing therapeutic development for AD. Indeed, cerebrovascular disease is a risk factor that deserves attention for AD [150]. APOA-I is effective in protecting against several vascular damages [151,152,153]. The strong vascular protective function may contribute to alleviating phosphorylated tau toxicity independently of PTMs and the clearance of pathological tau. Taken together, although APOA-I can modulate various kinases or enzymes in tau-phosphorylation and truncation within the CNS, the evidence for its direct impact on tauopathy remains limited. This is in contrast to its well-established role in mitigating Aβ toxicity. Considering that cognitive impairment in AD is more strongly associated with tau pathology than with Aβ deposition, further research is needed to elucidate APOA-I’s direct influence on tau pathogenesis and its potential to block Aβ-induced tauopathy in AD models.

### 3.3. APOA-I and Microglia

Microglia are resident macrophages in the CNS, which continuously surveil and regulate the brain microenvironment. Under normal physiological status, microglia are responsible for phagocytosing and degradation of metabolic products and are involved in regulating energy metabolism and neural activity in conjunction with neurons [154]. Microglia play a dual role in the development of AD. On the one hand, they are capable of engulfing and clearing Aβ and tau proteins, thereby limiting the spread of these pathological substances and potentially exerting protective effects in the early stages of the disease. On the other hand, when chronically stimulated by pathological deposits, these cells may become dysfunctional, shifting their function to promote neuroinflammation, accelerate the propagation of Aβ and tau, and ultimately contribute to neurodegenerative changes [155].

Wu et al. [156] recently reported that lipid droplets (LDs) accumulated in microglia and border-associated macrophages (BAMs) impaired phagocytosis function and aggravated Aβ plaque formation. Lower LDs in microglia and BAMs enhanced the efferocytosis capacities to reduce Aβ deposition. Intracellular LDs are formed by cholesteryl ester (CE), which is generated by the catalysis of free cholesterol by acyl-CoA/cholesterol acyltransferase (ACAT) [157]. Given that CNS cholesterol homeostasis depends on APOA-I-mediated efflux, reduced APOA-I levels may initiate LD accumulation in microglia, subsequently impairing their efferocytic function. Another study focusing on neuropathic pain indicated that APOA-I-binding proteins (AIBPs), ABCA1 and ABCG1, play a critical role in suppressing the activation of Toll-like receptor 4 (TLR4) and microglia-driven neuroinflammation via a cholesterol-dependent mechanism [110]. Similarly, AIBPs maintained microglial mitochondrial dynamics and alleviated TLR4-triggered neuroinflammation and further ameliorated neuronal injury in APP/PS1 transgenic mice [111]. Lewis TL et al. [112] designed an APP/PS1/APOA-I triple transgenic mice model and found that overexpressing the APOA-I gene significantly alleviates cerebral amyloid angiopathy (CAA) pathology and microglia activation. This model contained a 2-fold increase in plasma HDL levels and showed improved cognitive function compared with APP/PS1 mice. Furthermore, both oral administration of APOA-I mimetic peptide and intravenous treatment with human recombinant APOA-I Milano ameliorated AD pathology and microglia-derived neuroinflammation [113,114]. Interestingly, a recent study reported that 5xFAD transgenic mice fed a high-fat diet (HFD) showed higher serum HDL and APOA-I levels compared with mice fed a chow diet (CD). Correspondingly, the reactivity of astrocytes and microglia of HFD mice decreased, and the AD pathology of HFD mice was alleviated [158]. Despite the metabolic characteristics remaining controversial, this study still proved that APOA-I is negatively correlated with the inflammatory phenotype activation of microglia [158]. In general, APOA-I is significant for regulating microglia activation via maintaining brain cholesterol homeostasis. Supplemental APOA-I presents anti-neuroinflammation value for neurodegenerative diseases.

### 3.4. APOA-I and Astrocytes

Astrocytes are the most abundant cell type of the CNS and outnumber neurons by over fivefold, playing a crucial role in maintaining and supporting synaptic activity, neuronal metabolism, and the clearance of metabolic substances [159]. Metabolic dysfunction in astrocytes induces brain homeostasis disorder, which contributes to the development of AD pathology [160]. In turn, the AD pathology aggravates damage in astrocytes [161]. Hence, maintaining the homeostasis of astrocytes is significant to control AD progression.

The neurotoxic A1 reactive phenotype astrocytes in AD patients promote neuroinflammation and neurodegenerative pathology [162]. Remarkably, APOA-I was found to maintain the physiological function of astrocytes in several AD mouse models. The APP/PS1/APOA-I triple transgenic mice discussed above also showed inhibition of astrocyte overactivation [112]. Meanwhile, intravenous treatment with human recombinant APOA-I Milano also reversed the activation of astrocytes and ameliorated neuroinflammation in the APP23-transgenic mouse model [114]. A prominent investigation manifested that the APOE ε4 genotype, characterized by impaired lipid-carrying function, resulted in an abundant accumulation of cholesterol in astrocytes, exacerbating tau pathology in a tauopathy mouse model (P301S). Treatment with the liver X receptor (LXR) small-molecule agonist GW3965 upregulated ABCA1 expression in astrocytes, effectively cleared abnormal cholesterol accumulation, and alleviated tau pathology [163]. Although this study did not directly elucidate the role of APOA-I in this therapeutic approach, it is well-known that GW3965 effectively increases the levels of APOA-I within the CNS [164]. Furthermore, the binding of APOA-I to ABCA1 is a critical step for astrocytes to clear cholesterol [165]. Therefore, the protective effect observed in this study, achieved by using the LXR small-molecule agonist GW3965 and overexpressing ABCA1 in astrocytes without altering the APOE ε4 genetic background, may be mediated by the cholesterol reverse transport function from APOA-I. Actually, in addition to astrocytes, APOE ε4 also disrupted the lipid metabolism ability of microglia and oligodendrocytes, leading to the accumulation of lipid molecules, especially cholesterol [166,167]. Taken together, restoring lipid homeostasis presents a future treatment strategy for AD patients carrying the APOE ε4 gene, and APOA-I may hold potential therapeutic value.

### 3.5. APOA-I and Oligodendrocytes

Oligodendrocytes are supportive glial cells of the CNS, which are primarily responsible for the formation and maintenance of myelin sheaths, thereby promoting signal transmission between neurons [168]. Recent studies suggest that the disorder of oligodendrocytes leading to white matter loss or demyelination is related to AD [167,169]. In addition, oligodendrocytes directly secrete BACE1 (β-secretase) to participate in the cleavage of APP [170]. Thus far, the links between oligodendrocytes and AD are little known compared with astrocytes and microglia.

Joel W. Blanchard et al. [167] indicated that APOE ε4 significantly modifies signaling pathways related to cholesterol homeostasis and transportation, leading to abnormal cholesterol accumulation in oligodendrocytes, resulting in impairment in myelination processes and the electrical activity of neurons. Extensive accumulation of lipids derived from myelin metabolism promoted microglia and macrophages to a disease-promoting phagocyte phenotype [171]. Recently, a study indicated that APOA-I mimetic peptide 5A promoted myelin debris clearance and degradation to enhance remyelination via activating the ABCA1-JAK2-STAT3 signaling pathway in the CNS [115], confirming that APOA-I improves remyelination by enhancing lipid efflux in myelin. Similarly, another APOA-I mimetic, D-4F, is capable of clearing myelin debris and protecting for white matter damage [116,117]. Remarkably, J. K. Boyles et al. [118,119] present an alternative perspective on the interaction between APOA-I and oligodendrocytes. They propose that axonal regeneration requires cholesterol stores supplied by APOE, APOA-I, and macrophages that are recruited around the myelin. The lipid transport mediated by APOA-I provides the necessary substances for oligodendrocyte myelination. In conclusion, despite oligodendrocyte damage not being specific to AD, an increasing amount of evidence indicates that demyelination injury and impaired myelin regeneration are widespread in AD. APOA-I, on the one hand, is capable of clearing the debris and accumulated lipids during demyelination, thereby maintaining the stability of the CNS. On the other hand, APOA-I is responsible for transporting lipids for myelin regeneration, playing a crucial protective and supportive role in maintaining oligodendrocyte homeostasis.

## 4. APOA-I Is Associated with Multiple Risk Factors for AD

**Figure 5:**
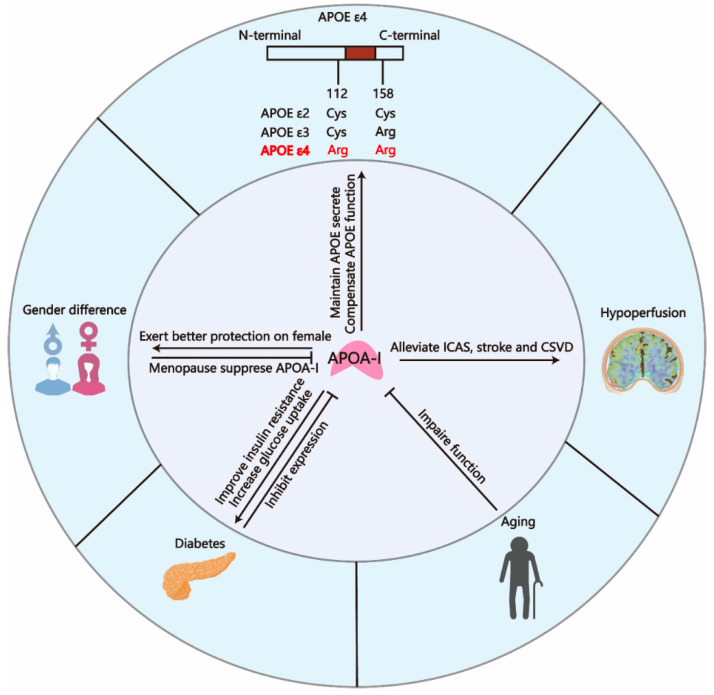
The mutual interactions between APOA-I and AD risk factors. In carriers of the APOE ε4 allele, lipid transport and Aβ clearance functions in the CNS are impaired. Given that APOA-I shares functional similarities with APOE, it can serve as a complementary treatment for APOE ε4 carriers [163,164,165,166]. Women are more susceptible to AD, while the protective effects of APOA-I are also more pronounced in healthy females [172,173,174]. The decline in APOA-I levels during perimenopause aligns with the increased incidence of dementia in perimenopausal women [175]. Cerebral hypoperfusion is a significant risk factor for AD, and APOA-I provides notable protection against conditions that induce cerebral hypoperfusion, such as internal carotid stenosis (ICS), cerebral small vessel disease (CSVD), and stroke [55,176,177]. Diabetes-related metabolic dysfunction plays a critical role in accelerating AD pathology during the preclinical stage. APOA-I significantly improves metabolic impairments related to cognition, such as insulin resistance and impaired glucose tolerance [178,179,180]. Aging, as an irreversible risk factor for AD, leads to a decline in the functionality of APOA-I and HDL-C. As a compensatory mechanism, APOA-I expression increases in the elderly, but its cholesterol transport capacity is markedly reduced, making it a potential diagnostic marker for late-onset AD [181,182,183].

Multiple risk factors are implicated in the accelerated progression or onset of AD, encompassing both non-modifiable factors (e.g., aging, sex, APOE alleles) and modifiable conditions (e.g., hypertension, diabetes, cerebrovascular diseases). Emerging research indicates that APOA-I exhibits both diagnostic potential and therapeutic promise for these risk categories. This section synthesizes current evidence linking APOA-I to AD risk factors, providing insights for developing APOA-I-based strategies in early diagnosis and preventive therapeutics. The link between APOA-I and AD risk factors is presented in Figure 5.

### 4.1. APOA-I and Gender Difference

The risk of developing AD exhibits gender differences, with women constituting approximately 70% of the affected population. In women, the progression of the disease occurs at a rate nearly three times faster than in men and is associated with a broader spectrum of cognitive symptoms [184]. Furthermore, the incidence rate of AD in women escalates significantly during the perimenopausal period [185,186].

The cause of gender difference is not fully understood. Our recent research has confirmed that the markedly elevated levels of follicle-stimulating hormone (FSH) during the perimenopausal period, along with the presence of the APOE ε4 allele, constitute significant risk factors contributing to the heightened susceptibility to AD in women [187,188]. Another study published recently found that disrupted lipid metabolism is attributed to increased susceptibility of females to AD [189]. As the most crucial components regulating lipid homeostasis, APOA-I and HDL-C showed higher concentrations in females than males [172]. The sex-specific mechanism of APOA-I expression is not fully understood. It is proposed that women may exhibit a higher rate of HDL-APOA-I synthesis compared with men, which is associated with an enhanced cholesterol efflux capacity [173]. Additionally, menopause contributed to lowering APOA-I levels [175], which is positively related to cognitive decline. More importantly, decreased HDL-C-APOA-I levels are more strongly associated with systemic damage in females compared with males [174]. Given that several studies showed that APOA-I decreased in AD and dementia patients [83,84,88,190], APOA-I may mediate the gender difference in AD prevalence. Research on gender differences in APOA-I levels may hold particular significance for the prevention and treatment of AD in women.

### 4.2. APOA-I and Aging

Based on the age at which symptoms first appear, AD is classified into two types: early-onset AD (EOAD) and late-onset AD (LOAD), with the demarcation established at 65 years [191]. The likelihood of developing AD increases substantially with advancing age, particularly LOAD. Specifically, approximately 10% of individuals aged 65 and older are affected by AD. As a result, aging is recognized as the inevitable risk factor for the disease [192]. The impact of aging on AD mainly includes the following aspects [193]: (1) Genomic instability: Aging exacerbates genomic instability in neurons, thereby increasing their susceptibility to AD [194]. This process is primarily mediated by age-related declines in antioxidant capacity and abnormalities in the cerebrovascular microenvironment. (2) Telomere shortening: Aging-induced telomere shortening further aggravates DNA damage, promoting the pathological progression of AD [192]. (3) Epigenetic alterations: Aging leads to epigenetic changes, such as DNA methylation, histone modifications, and dysregulation of non-coding RNA mechanisms, which disrupt systemic homeostasis [195]. (4) Loss of proteostasis: Aging-induced imbalance in protein homeostasis is a critical factor in the pathogenesis of AD, resulting in the accumulation and impaired clearance of abnormal proteins, finally inducing metabolic dysfunction [196].

Interestingly, a large-scale prospective study involving 3.3 million participants demonstrated that elevated HDL-C levels are associated with an increased risk of dementia in both elderly men and women. Furthermore, this association appears to be independent of traditional dementia risk factors and genetic influences, contrary to the traditional view that HDL-C has a protective effect [197]. The potential reason for this paradox is that aging leads to the degradation of HDL functionality. Alexis Nasr et al. [181] and Meiyuzhen Qi et al. [182] found that the size rather than quantity of HDL metrics was associated with future cognitive function of women, and the number of larger HDL particles related to cognitive dysfunction increased in women with aging. The larger size indicates an accumulation of cholesterol and phospholipids, reflecting impaired lipid-carrying functionality (mainly mediated by APOA-I) of HDL. These results demonstrate that the functionality of HDL gradually deteriorates with aging, accompanied by impaired lipid transport capacity. Another study reported that HDL isolated from elderly subjects exhibited impaired cholesterol uptake and antioxidant capacity compared with that from younger subjects [183]. In summary, among the elderly, the quantity of HDL is no longer the primary factor influencing cognition, as the functionality of HDL is compromised by aging. Elevated HDL levels and larger HDL particle size indicate impaired lipid-carrying capacity in the elderly. However, although APOA-I constitutes the majority of HDL-C (over 75%), the roles of other components within HDL-C, such as APOA-II and APOA-J, should not be neglected [49]. In conclusion, the impact of aging on APOA-I primarily manifests as functional impairment, which is associated with dementia and AD.

### 4.3. APOA-I and APOE ε4

APOA-I and APOE share nearly identical functions, operating across various organs. APOE ε4 is a strong risk determinant of LOAD. Possessing a single APOE ε4 allele elevates the risk of developing LOAD by 3 to 4 times, while having two alleles raises the risk by 9 to 15 times [198,199,200]. In the CNS, APOE is predominantly produced by astrocytes, microglia, vascular mural cells, and choroid plexus cells, with minimal expression observed in stressed neurons under certain conditions [14]. Following its secretion from these cells, cholesterol and phospholipids are transferred to newly synthesized APOE by the cell surface ATP-binding cassette transporters ABCA1 and ABCG1, facilitating the formation of HDL particles, which is similar to the role of APOA-I in the periphery [201,202]. The most extensively acknowledged aspect of the relationship between the APOE genotype and AD is its correlation with Aβ deposition. In neurons derived from human embryonic stem cells and induced pluripotent stem cells (iPSCs), APOE ε4 stimulated greater Aβ production and secretion compared with other isoforms [203,204]. Additionally, APOE ε4 facilitated the creation of Aβ fibrils by speeding up the early stages of Aβ peptide seeding or nucleation [205]. Furthermore, the Aβ clearance within the brain seemed slower in APOE ε4 compared with other alleles [206,207,208]. Notably, the mechanism of Aβ pathology induced by APOE ε4 closely aligns with the functions of APOA-I discussed in the “APOA-I and Aβ” section, where APOA-I inhibits APP cleavage and mediates Aβ clearance. This suggests that APOA-I may hold significant therapeutic potential for AD patients carrying the APOE ε4 genotype, particularly given that genetic predisposition is an inevitable risk factor. Supporting this theory, the study by Alexandra Litvinchuk et al. [163] provided evidence demonstrating that the LXR agonist GW3965 ameliorated neurodegenerative pathology in APOE ε4 genotype mice by addressing lipid dysregulation in the brains of P301 transgenic mice. Notably, GW3965 is known to significantly increase APOA-I expression in the CNS, thereby alleviating AD-related pathology [164]. Indeed, lipid transport, which is another shared function of APOA-I and APOE, has recently been reported as a key mechanism through which APOE ε4 contributes to the pathogenesis of AD [166]. APOE ε4 triggers lipid metabolic dysfunction in astrocytes and microglia [209]. Additionally, Aβ deposition, driven by lipid dysregulation, further exacerbates lipid synthesis in microglia, leading to hyperphosphorylation of tau protein and neuronal apoptosis in patients containing APOE ε4/ε4 isoform rather than APOE ε3/ε3 [20]. The impaired lipid transport in patients carrying the APOE ε4/ε4 isoform is associated with ABCA1 disorder. Analysis of brain tissue samples from AD patients carrying APOE ε4 revealed that ABCA1 is sequestered in lysosomes, preventing it from performing its cholesterol transport function. This disruption leads to insufficient generation of HDL [210]. Given that APOA-I and APOE share common receptors in the CNS (such as ABCA1, SR-BI, and ABCG1), these findings further highlight the potential for APOA-I to serve as a substitute therapy for APOE functional deficiency in AD patients carrying the APOE4 genotype.

### 4.4. APOA-I and Diabetes

Research has further indicated that individuals with diabetes frequently exhibit cognitive deficits resembling those seen in the initial phases of AD [211]. The prevalence of impaired glucose tolerance and diabetes in AD patients is 80% higher than in the general population [212]. The shared pathological features between diabetes and AD, including impaired glucose tolerance, insulin resistance, oxidative stress, neuroinflammation, and cerebrovascular dysfunction, are closely associated with hallmark AD pathologies (Aβ deposition and neurofibrillary tangles). This overlap has led some researchers to propose the concept of “type 3 diabetes” (brain-specific diabetes) [213,214,215,216].

Notably, the two characteristic symptoms of diabetes are surprisingly related to the low expression of APOA-I [217,218]. More evidence can be seen in the research that the hypoglycemic drug metformin failed to improve insulin resistance and increase glucose uptake into skeletal muscle in APOA-I^−^/^−^ mice [178]. Nevertheless, APOA-I supplementation significantly rescued impaired glucose tolerance and reduced fasting hyperglycemia in APOA-I^−^/^−^ mice [179]. The same therapeutic effect has also been observed clinically. Following a single administration of artificially engineered discoidal HDL particles, which consist of APOA-I briefly combined with soybean phospholipids, a temporary rise in plasma HDL concentration was noted. This intervention led to decreased levels of APOA-I, insulin, and blood glucose in individuals diagnosed with type 2 diabetes mellitus (T2DM) [180]. Chronic enhancement of HDL cholesterol and APOA-I concentrations over the long term through the inhibition of cholesteryl ester transfer protein (CETP) activity has also been demonstrated to enhance glycemic regulation in individuals with T2DM [219,220]. The mechanisms by which APOA-I alleviates diabetes are diverse and multifaceted. APOA-I significantly activated the insulin receptor substrate-1/phosphatidyl inositol 3-kinase/Akt (PI3K-activated serine-threonine kinase) substrate (IRS-PI3K/Akt) pathway to improve insulin resistance and impaired glucose uptake in adipose tissue and skeletal muscle via increasing the translocation of glucose transporter type 4 (GLUT4) [221,222,223]. Additionally, a clinical investigation demonstrated that a single administration of discoidal reconstituted HDL enhanced glucose uptake into skeletal muscle in individuals with T2DM, mediated through mechanisms dependent on calcium/calmodulin-dependent protein kinase (CaMKK) and AMP-activated protein kinase (AMPK) [180]. It is widely recognized that the inhibition of the IRS-PI3K/Akt pathway, resulting from diabetes-related damage, is one of the mechanisms that exacerbates AD [224,225]. Additionally, GLUT4, a critical receptor responsible for glucose transport across the BBB and its uptake by neurons, is impaired in AD [226,227]. This impairment leads to reduced glucose uptake in the brain, which is a hallmark metabolic alteration observed in AD [228]. Furthermore, as a key regulator of energy metabolism, AMPK is also suppressed in the brains of AD patients, leading to impaired glucose utilization in neurons [229]. The shared pathological mechanisms between diabetes and AD, along with the protective effects of APOA-I against these impairments, suggest that APOA-I may serve as a potential therapeutic target for mitigating the exacerbation of AD induced by diabetes. More importantly, APOA-I protected the mouse MIN6 insulinoma cell line (simulating pancreatic β-cells) against damage by inhibiting the expression of C/EBP (CCAAT/enhancer-binding protein) homologous protein [230,231]. Our preliminary studies have demonstrated that CEBP/β in neurons can exacerbate the pathological progression of AD by cleaving APP and tau through the activation of asparagine endopeptidase (AEP, also known as δ-secretase) [122,187,188]. Nevertheless, these mechanisms, including insulin resistance, impaired glucose tolerance, and the activation of C/EBPβ, contribute to the pathology of AD only when they occur within the CNS [232,233,234]. Current research on APOA-I in relation to these mechanisms has been confined to peripheral tissues, and it remains unclear whether the protective effects of APOA-I against these associated impairments also operate within the CNS. Given the significant role of diabetes-induced metabolic dysfunction in promoting AD pathology, it is crucial to investigate whether the protective effects of APOA-I against metabolic damage in diabetes also extend to the CNS. This exploration is particularly important for developing therapeutic interventions targeting brain-specific diabetes.

### 4.5. APOA-I and Cerebrovascular Diseases

Historically, vascular dementia (VD) and AD have been regarded as two relatively distinct types of dementia, classified based on their different pathological features and clinical symptoms [235]. Nevertheless, as research into dementia has advanced, scholars have discovered that the pathologies of AD and VD often coexist. Large-scale postmortem brain studies have revealed that more than 1/3 of dementia patients exhibit severe cerebral arteriosclerosis, with an even higher prevalence of such lesions observed in AD cases [150]. Additionally, Aβ deposition has been detected in over 30% of elderly non-AD patients [236]. These findings indicate that AD and VD frequently occur together and mutually influence each other. Consequently, the concept of mixed dementia has been proposed to describe dementia cases characterized by the coexistence of multiple pathological types. In clinical practice, mixed dementia accounts for more than ~70% of cases [237,238]. Consequently, the concept of the neurovascular unit (NVU) has been introduced and is now recognized as a key functional unit mediating the pathology of AD. The NVU, composed of neurons, glial cells, and blood vessels, serves as a critical structure for maintaining the stability of the brain’s microenvironment [79]. When the structural and functional integrity of the NVU is compromised, it disrupts the brain’s energy supply and microenvironmental homeostasis, thereby influencing the pathophysiological processes and cognitive functions associated with AD. Cerebral hypoperfusion has been extensively studied as a high-risk factor in the early stages of AD development. Reduced cerebral blood flow is primarily caused by cerebral atherosclerosis, cerebral small vessel disease (CSVD), and ischemic stroke, and hypoperfusion can lead to AD-like neuronal death and neurotoxicity, which promote memory loss [239]. Furthermore, hypoperfusion activates β-secretase and γ-secretase, exacerbating the production of Aβ [240,241,242,243]. Additionally, hypoperfusion induces the activation of tau kinases, such as mitogen-activated protein kinase (MAPK), also known as extracellular signal-regulated kinase (ERK), through hypoxia-inducible factor 1-alpha (HIF-1α), thereby intensifying tau phosphorylation [244]. Therefore, targeting the underlying causes of neurovascular unit (NVU) dysfunction is particularly crucial for the early prevention and treatment of AD, as reduced cerebral blood flow occurs before the onset of clinical symptoms of AD.

Elevated levels of APOB and reduced levels of APOA-I are among the most significant indicators for predicting intracranial atherosclerotic stenosis (ICAS) [55,176] since APOB and APOA-I serve as the core components of low-density lipoprotein (LDL) and HDL, respectively. LDL and HDL play opposing roles in the development and progression of atherosclerosis. LDL is responsible for transporting cholesterol from the liver to peripheral tissues, and elevated LDL levels lead to the infiltration and accumulation of LDL particles within the subendothelial space of arterial walls, which trigger inflammatory responses and recruit macrophages, leading to the formation of foam cells. In contrast, HDL exerts protective effects against atherosclerosis by collecting excess cholesterol from arteries, exhibiting anti-inflammatory properties, and increasing the stability of atherosclerotic plaques [245]. Apart from macrovascular dysfunction, APOA-I and HDL-C also exhibited an inverse relationship with the severity and occurrence of CSVD, manifested by a negative correlation with small vessel stroke, silent brain infarction, and white matter hyperintensity, especially for females [174,177]. In summary, the protective role of APOA-I against vascular diseases that cause cerebral hypoperfusion is well established. APOA-I can serve as a protective agent during the phase of reduced cerebral blood flow that occurs before the onset of clinical symptoms of AD. These findings have profound implications for the principles of early diagnosis and early intervention in AD.

## 5. APOA-I Based Therapy

APOA-I has been established as a crucial therapeutic target in cardiovascular medicine. To date, multiple therapeutic agents mimicking APOA-I function have been developed, including APOA-I mimetic peptides, mutated variants of APOA-I, and full-length APOA-I. Notably, most of these agents have demonstrated significant therapeutic efficacy in preclinical AD studies (Table 1). In this section, we will summarize the therapeutic applications of APOA-I-based approaches in AD research.

The first APOA-I mimetic peptide, 18A, synthesized by Anantharamaiah et al., comprises 18 amino acids [250]. Subsequently, Yancey et al. developed a modified peptide designated as 2F by blocking the end of 18A with phenylalanine (F) residues [251]. The progressive addition of phenylalanine residues (from 2F to 7F, as denoted by the numeric prefix) demonstrated a positive correlation with both lipid-binding capacity and hydrophobic character. The APOA-I mimetic peptide 4F is widely studied in AD pathology. Previous studies have demonstrated that the 4F peptide enhances both the secretion and lipidation of APOE in microglia and astrocytes. Furthermore, 4F ameliorates Aβ-induced suppression of APOE functionality through ABCA1 activation, thereby preserving APOE activity [246,252]. Further investigations demonstrated 4F-mediated Aβ trafficking modulation, showing its capacity to bind plasma Aβ, thereby reducing the CNS influx while simultaneously promoting the efflux of cerebral Aβ [15]. The latest preclinical studies revealed that 4F treatment achieves a statistically significant reduction in cerebral amyloid angiopathy burden and Aβ-mediated cytotoxic effects in AD transgenic mice [248,249]. Additionally, the oral peptide synthesized with D-amino acids, named D-4F, also ameliorates Aβ burden and improves cognitive function in AD transgenic mice [113]. In addition to APOA-I peptides, the APOA-I Milano mutation, containing enhanced anti-inflammatory, anti-atherosclerotic properties and a more potent ABCA1-dependent cholesterol efflux mediator, has also shown promising therapeutic potential for AD treatment. The human recombinant APOA-I Milano showed more powerful neuroprotection against Aβ (1–42)-toxicity and suppression of neuroinflammation from glial cells in APP23-transgenic AD mouse [114]. Furthermore, APOA-I Milano promotes Aβ40 removal from the brain [134]. Exogenous APOA-I supplementation has demonstrated favorable therapeutic effects in preclinical studies. Parallelly, endogenous APOA-I augmentation achieved through a novel APP/PS1/APOA-I triple-transgenic mouse model designed by Lewis et al. similarly ameliorates AD pathology. The APP/PS1/APOA-I triple-transgenic mouse model exhibits higher levels of serum HDL and improved cerebral amyloid angiopathy and neuroinflammation compared with the APP/PS1 double-transgenic model [112]. In brief, while APOA-I-based therapies have been widely used in cardiovascular diseases and the atherosclerosis area, application in neurodegeneration requires further clinical trials. Notably, multiple APOA-I mimetics and drugs have demonstrated clinical safety profiles in cardiovascular trials [49]. Coupled with substantial preclinical evidence in AD models, this significantly reduces translational barriers for their repurposing in AD therapeutics.

## 6. APOA-I and Other Neurodegenerative Diseases

Neurodegenerative diseases share several common pathogenic mechanisms, including oxidative stress, metabolic dysregulation, and epigenetic alterations. This section synthesizes existing evidence on the associations between APOA-I and other major neurodegenerative disorders, aiming to establish a comprehensive foundation for APOA-I’s therapeutic applications in this field.

Parkinson’s disease (PD) ranks as the second most common neurodegenerative disorder. PD demonstrates strong pathological links to α-synuclein (α-syn) mutations [253]. Emerging evidence implicates brain cholesterol metabolism and lipoprotein dynamics in modulating α-syn activity. Clinically, reduced serum concentrations of both APOA-I and HDL cholesterol correlate significantly with early-onset PD manifestation and elevated disease susceptibility [254,255,256]. Two recent randomized controlled trials have demonstrated that statin therapy may reduce PD risk by elevating APOA-I and HDL levels [257,258]. One proposed mechanism by which APOA-I exerts neuroprotective effects in PD involves the binding of α-syn into HDL particles, thereby preventing α-syn aggregation through conformational remodeling [259]. In parallel, APOA-I’s potent antioxidant capacity may mitigate oxidative damage in dopamine transporter (DAT) [260,261]. The inhibitory effect of APOA-I on α-syn aggregation shares mechanistic parallels with its suppression of Aβ fibrillization. Elucidating the precise regulation of APOA-I’s interactions with these pathological proteins represents a critical frontier in neurodegenerative disease research.

Frontotemporal dementia (FTD) is a major neurodegenerative disorder causing dementia that is distinct from AD. Unlike AD, FTD typically manifests before the age of 65 and can be classified into behavioral variant FTD (bvFTD), semantic variant primary progressive aphasia (svPPA), and nonfluent/agrammatic variant PPA (nfvPPA) [262]. The hallmark feature of FTD is atrophy of the frontal and temporal lobes; however, the exact etiology remains unclear. Some theories suggest that brain atrophy may be linked to lipid loss in brain tissue [263]. Kim et al. [263] reported a marked reduction in the levels of APOA-I and APOA-II in FTD patients. Additionally, decreased HDL, elevated triglycerides, and insulin resistance have been identified as metabolic characteristics in FTD [264]. Although emerging evidence indicates that lipid dysregulation may contribute to FTD pathogenesis or metabolic dysfunction, comprehensive research in this area remains limited. Notably, the lipidomic profiles of FTD differ from those of AD, suggesting potential biomarkers for differentiating between these two neurodegenerative disorders.

Amyotrophic lateral sclerosis (ALS) is a fatal neurodegenerative disorder characterized by progressive muscle weakness and respiratory dysfunction, typically leading to mortality within 3–5 years of onset. The etiology of ALS remains poorly understood, though genetic predisposition has been the most extensively studied component [265]. However, due to the uncertain pathogenic role of many identified gene variants, targeted therapies derived from genetic insights remain limited. Consequently, a multi-system comparative analysis of biomarkers between amyotrophic lateral sclerosis patients and healthy populations represents a fundamental strategy for elucidating disease pathogenesis and developing diagnostic and therapeutic approaches. Notably, lipidomic characterization has become a predominant research direction in contemporary ALS investigations [266]. Current research presents inconsistent findings regarding lipid profiles in ALS. Two studies utilizing Mendelian randomization analysis demonstrated that elevated LDL-C levels may increase ALS risk [266,267]. However, it should be noted that these conclusions might be influenced by population-specific genetic variations inherent to the methodology. Interestingly, a meta-analysis identified that higher triglyceride levels were associated with improved survival in ALS patients [268]. In clinical observational studies, the Swedish AMORIS cohort with over 20 years of follow-up revealed elevated levels of LDL-C, HDL-C, APOB, and APOA during early ALS stages. Notably, the LDL-C/HDL-C and APOB/APOA ratios demonstrated superior diagnostic specificity when measured 10 years post-ALS onset [269]. These findings were corroborated by a multicenter study involving five research centers, which established a positive correlation between high HDL-C levels and ALS pre-diagnosis [270]. These large-scale, longitudinal studies collectively highlight the significant elevation of HDL-C during both pre-symptomatic and early disease phases, providing crucial insights for future ALS research directions. Additionally, Barros et al. [271] reported decreased serum copper levels in ALS patients, which showed a correlation with reduced HDL-C concentrations. Collectively, emerging evidence suggests stage-dependent fluctuations in lipidomic signatures throughout ALS disease progression. Comprehensive longitudinal investigations incorporating real-world data are critically needed to characterize this temporal metabolic shift, potentially paving the way for developing disease-modifying interventions targeting specific phases of ALS pathogenesis.

## 7. Conclusions

Recent research has increasingly focused on disease-modifying treatment (DMT) as the understanding of AD has advanced [272]. Despite significant progress, DMT requires strict patient eligibility criteria, while more broadly applicable treatments remain to be developed. APOA-I, known for its robust lipid transport and antioxidant capabilities, has been widely utilized in the treatment of cardiovascular diseases and atherosclerosis. Since earlier research indicated that APOA-I is not expressed locally in the CNS (as APOA-I mRNA is undetectable [29]) and due to the lack of studies on the transport of APOA-I to the CNS, its research and application were largely confined to the peripheral system for an extended period. With the increasing reports of the correlation between AD and APOA-I in clinical patients, as well as preclinical studies on the transport of APOA-I to cross the BBB and BCSFB, this multifunctional apolipoprotein, which integrates lipid metabolism regulation, vascular protection, neuroprotection, anti-inflammatory, and antioxidant properties, has begun to garner significant attention. As summarized above, multiple studies have confirmed that APOA-I levels are reduced in the CSF and brain of AD patients. Moreover, the impairment in function and decrease in levels of APOA-I are associated with AD risk factors, including diabetes, cerebrovascular diseases, and aging. Notably, the protective effects of APOA-I are more pronounced in women, which aligns with the epidemiological characteristic that AD is more prevalent in females. In other words, various AD risk factors exert an inhibitory effect on APOA-I, suggesting that APOA-I may exhibit high sensitivity in the early diagnosis of AD. It could serve as a warning indicator during the preclinical stage before the progression to clinical cognitive dysfunction. Furthermore, APOA-I is directly linked to the pathology of AD. It alleviates the cleavage of APP, promotes the clearance of Aβ, and inhibits kinases associated with tau protein phosphorylation, thereby mitigating the AD-like pathology. Current perspectives suggest that balancing lipid metabolism in the CNS is a crucial approach to treat AD, and various APOA-I mimetics and activators have been shown to modulate lipid metabolism in glial cells, alleviating AD pathology. More importantly, APOA-I and APOE perform highly similar functions and share overlapping cellular receptors in the CNS. Additionally, APOE is synthesized centrally by the glial system, while peripheral APOE does not exert its functions in the CNS [273]. Therefore, APOA-I supplementation therapy holds significant promise for APOE ε4 carriers. In summary, the incorporation of APOA-I into existing biomarker panels may improve diagnostic sensitivity and specificity for AD, and early therapeutic interventions using APOA-I-targeted agents may represent a promising advancement in AD management. Additionally, APOA-I is relevant throughout the entire stage of AD, and a substantial body of research has already demonstrated its protective effects against neurodegenerative diseases, making it a key substance that indicates early diagnosis and treatment for AD.

## Figures and Tables

**Table 1:** The application of APOA-I in AD models.

APOA-I Modulation	Model	Effects	Reference
Peptide D-4F + statin	APP/PS1 transgenic mice	Improves cognitive function; ameliorates Aβ burden, suppresses microglial and astrocytic activation, and downregulates pro-inflammatory cytokines IL-1β and TNF-α	[113]
Peptide 4F	Human astrocytes; primary mouse astrocytes and microglia	Enhances glial APOE secretion and lipidation, attenuates Aβ-induced APOE dysfunction, activates ABCA1 to maintain APOE functionality	[246]
D-4F labeled with ^125^I	B6SJLF1/J mice injected with Aβ42 and Aβ40; hCMEC/D3 cell line	Increases brain efflux of Aβ42, decreases brain influx of Aβ42 but not Aβ40, decreases Aβ42 accumulation in hCMEC/D3	[15]
Peptide 4F	In vitro binding interaction between Aβ fragments and 4F peptide	Slows down the aggregation kinetics of Aβ (1–42), constrains the structural plasticity of Aβ	[247]
Peptide 4F	APP/PS1 transgenic mice	Decreased Aβ42-induced p38 activation in BBB endothelial cells	[248]
Peptide 4F	Tg-SwDI mouse	Attenuated CAA-associated microgliosis, reduced CAA, mitigated inflammation from vascular smooth muscle cells	[249]
Human APOA-I gene knock-in	APP/PS1/AI triple Tg mice	Exhibits a 2-fold increase in plasma HDL cholesterol levels, reduces cerebral amyloid angiopathy, decreases Aβ-induced neuroinflammation	[112]
Purified human APOA-I extraction and lipidation induce	In vitro binding interaction between purified human APOA-I and Aβ_1–42,_ hCMEC/D3 cell line	The initially lipidated human APOA-I demonstrates superior BBB penetrability and most effectively mediates Aβ efflux	[23]
Human recombinant APOA-I Milano	APP23-transgenic mouse	The APOA-I Milano shows more powerful neuroprotection and anti-inflammation ability compared to wild-type APOA-I	[114]
Human recombinant APOA-I Milano	APP23-transgenic mouse	Improves cognitive function and anxiety behavior, reduces Aβ40 in the brain and increases Aβ40 in CSF (promotes efflux), ameliorates endothelial damage	[134]

## Data Availability

All data are included in this manuscript.
